# A Literature Review of Word of Mouth and Electronic Word of Mouth: Implications for Consumer Behavior

**DOI:** 10.3389/fpsyg.2017.01256

**Published:** 2017-07-25

**Authors:** Nuria Huete-Alcocer

**Affiliations:** Economía Española e Internacional, Econometría e Historia e Instituciones Económicas, University of Castilla-La Mancha Albacete, Spain

**Keywords:** businesses, consumers, WOM, eWOM, communication

## Abstract

The rise and spread of the Internet has led to the emergence of a new form of word of mouth (WOM): electronic word of mouth (eWOM), considered one of the most influential informal media among consumers, businesses, and the population at large. Drawing on these ideas, this paper reviews the relevant literature, analyzing the impact of traditional WOM and eWOM in the field of consumer behavior and highlighting the main differences between the two types of recommendations, with a view to contributing to a better understanding of the potential of both.

## Introduction

Consumers increasingly use online tools (e.g., social media, blogs, etc.) to share their opinions about the products and services they consume (Gupta and Harris, 2010; Lee et al., 2011) and to research the companies that sell them. These tools are significantly changing everyday life and the relationship between customers and businesses (Lee et al., 2011).

The rapid growth of online communication through social media, websites, blogs, etc., has increased academic interest in word of mouth (WOM) and electronic word of mouth (eWOM) (e.g., Hennig-Thurau et al., 2004; Brown et al., 2007; Cheung and Thadani, 2012; Hussain et al., 2017; Yang, 2017). Specifically, the present paper will review the literature on how these two media have evolved, the main differences between them, and the degree to which they influence both businesses and consumers, now that they have become some of the most influential information sources for decision-making.

## Background

Word of mouth is one of the oldest ways of conveying information (Dellarocas, 2003), and it has been defined in many ways. One of the earliest definitions was that put forward by Katz and Lazarsfeld (1966), who described it as the exchanging of marketing information between consumers in such a way that it plays a fundamental role in shaping their behavior and in changing attitudes toward products and services. Other authors (e.g., Arndt, 1967) have suggested that WOM is a person-to-person communication tool, between a communicator and a receiver, who perceives the information received about a brand, product, or service as non-commercial. Likewise, WOM has been defined as communication between consumers about a product, service, or company in which the sources are considered independent of commercial influence (Litvin et al., 2008). These interpersonal exchanges provide access to information related to the consumption of that product or service over and above formal advertising, i.e., that goes beyond the messages provided by the companies and involuntarily influences the individual’s decision-making (Brown et al., 2007). WOM is widely regarded as one of the most influential factors affecting consumer behavior (Daugherty and Hoffman, 2014). This influence is especially important with intangible products that are difficult to evaluate prior to consumption, such as tourism or hospitality. Consequently, WOM is considered the most important information source in consumers’ buying decisions (Litvin et al., 2008; Jalilvand and Samiei, 2012) and intended behavior. For example, tourist satisfaction is of utmost importance because of its influence on behavioral intentions, WOM and purchasing decisions. In other words, overall satisfaction leads to the possibility of revisiting and recommending the destination (Sotiriadis and Van Zyl, 2013).

Similarly, previous research indicates that consumers regard WOM as a much more reliable medium than traditional media (e.g., television, radio, print advertisements, etc.) (Cheung and Thadani, 2012). It is thus considered one of the most influential sources of information about products and services (Lee and Youn, 2009). Users generally trust other consumers more than sellers (Nieto et al., 2014). As a result, WOM can influence many receivers (Lau and Ng, 2001) and is viewed as a consumer-dominated marketing channel in which the senders are independent of the market, which lends them credibility (Brown et al., 2007). This independence makes WOM a more reliable and credible medium (Arndt, 1967; Lee and Youn, 2009).

Today’s new form of online WOM communication is known as electronic word-of-mouth or eWOM (Yang, 2017). This form of communication has taken on special importance with the emergence of online platforms, which have made it one of the most influential information sources on the Web (Abubakar and Ilkan, 2016), for instance, in the tourism industry (Sotiriadis and Van Zyl, 2013). As a result of technological advances, these new means of communication have led to changes in consumer behavior (Cantallops and Salvi, 2014; Gómez-Suárez et al., 2017), because of the influence they enable consumers to exert on each other (Jalilvand and Samiei, 2012) by allowing them to obtain or share information about companies, products, or brands (Gómez-Suárez et al., 2017).

One of the most comprehensive conceptions of eWOM was proposed by Litvin et al. (2008), who described it as all informal communication via the Internet addressed to consumers and related to the use or characteristics of goods or services or the sellers thereof. The advantage of this tool is that it is available to all consumers, who can use online platforms to share their opinions and reviews with other users. Where once consumers trusted WOM from friends and family, today they look to online comments (eWOM) for information about a product or service (Nieto et al., 2014).

As a result of ICT, today consumers from all over the world can leave comments that other users can use to easily obtain information about goods and services. Both active and passive consumers use this information medium (eWOM). Individuals who share their opinions with others online are active consumers; those who simply search for information in the comments or opinions posted by other customers are passive consumers (Wang and Fesenmaier, 2004).

Electronic word of mouth also provides companies with an advantage over traditional WOM insofar as it allows them both to try to understand what factors motivate consumers to post their opinions online and to gauge the impact of those comments on other people (Cantallops and Salvi, 2014). However, consumers’ use of technology to share opinions about products or services (eWOM) can be a liability for companies, as it can become a factor they do not control (Yang, 2017). To counteract this, businesses are seeking to gain greater control of customers’ online reviews by creating virtual spaces on their own websites, where consumers can leave comments and share their opinions about the business’s products and services (Vallejo et al., 2015). By way of example, in the field of tourism, companies are starting to understand that ICT-enabled media influence tourists’ purchasing behavior (Sotiriadis and Van Zyl, 2013).

Understandably, companies view both types of recommendations – WOM and eWOM – as a new opportunity to listen to customers’ needs and adjust how they promote their products or services to better meet them, thereby increasing their return. A negative or positive attitude toward the product or service will influence customers’ future purchase intentions by allowing them to compare the product or service’s actual performance with their expectations (Yang, 2017).

In the field of consumer behavior, some previous studies (e.g., Park and Lee, 2009) have shown that consumers pay more attention to negative information than to positive information (Cheung and Thadani, 2012). For example, the customers most satisfied with a product or service tend to become loyal representatives thereof via positive eWOM (Royo-Vela and Casamassima, 2011), which can yield highly competitive advantages for establishments, businesses, or sellers, especially smaller ones, which tend to have fewer resources. Some studies have suggested that traditional WOM is the sales and marketing tactic most often used by small businesses.

Additionally, eWOM offers businesses a way to identify customers’ needs and perceptions and even a cost-effective way to communicate with them (Nieto et al., 2014). Today, eWOM has become an important medium for companies’ social-media marketing (Hussain et al., 2017).

### WOM vs. eWOM

While many authors (e.g., Filieri and McLeay, 2014) consider eWOM reviews to be electronic versions of traditional WOM reviews, this paper aims to summarize and explain the main differences between the two concepts (**Table 1**). The first such difference is credibility as an information source (Cheung and Thadani, 2012; Hussain et al., 2017), since it can influence consumers’ attitudes toward products or services (Veasna et al., 2013), for example, with regard to the purchase of tourism services, which are considered to be high-risk (Sotiriadis and Van Zyl, 2013). Luo et al. (2013) have suggested that the anonymity of online messages could have a negative effect on their credibility. In contrast, other studies (e.g., Hussain et al., 2017) have argued that consumers use eWOM more to reduce risk when decision-making. Likewise, eWOM tends to be more credible when the consumer using it has previous experience (Sotiriadis and Van Zyl, 2013).

**Table 1:** Differences between WOM and eWOM.

	WOM	eWOM
Credibility	The receiver of the information knows the communicator (positive influence on credibility)	Anonymity between the communicator and the receiver of the information (negative influence on credibility)

Privacy	The conversation is private, interpersonal (via dialogs), and conducted in real time	The shared information is not private and, because it is written down, can sometimes be viewed by anyone and at any time

Diffusion speed	Messages spread slowly. Users must be present when the information is being shared	Messages are conveyed more quickly between users and, via the Internet, can be conveyed at any time

Accessibility	Less accessible	Easily accessible


*Source: The author*.

Message privacy is another feature that sets the two media apart, since with traditional WOM information is shared through private, real-time, face-to-face dialogs and conversations. In contrast, information shared through eWOM is not private and can sometimes be seen by anonymous people who do not know each other. Furthermore, reviews can be viewed at various points in time (Cheung and Thadani, 2012). Indeed, because eWOM reviews are written, consumers and companies can check them at any time; this stands in contrast to traditional WOM, where once the message has reached the receiver, it tends to disappear.

Another salient difference between the two media is the speed of diffusion of the message; eWOM statements spread much faster than WOM statements because of where they are published, i.e., on the Internet (Gupta and Harris, 2010). Online platforms for sharing information (social media, websites, blogs, etc.) are what set eWOM apart from traditional WOM (Cheung and Thadani, 2012). First, they make the reviews accessible to more consumers (Cheung and Thadani, 2012; Sotiriadis and Van Zyl, 2013). Second, because they are written, they persist over time (Hennig-Thurau et al., 2004; Cheung and Thadani, 2012).

## Conclusion

This paper has reviewed the literature with a view to providing a clearer understanding of WOM and eWOM in the context of consumer information searches.

To this end, the review found that, in keeping with numerous studies, WOM is both the oldest medium for sharing opinions about products or services and the one most likely to influence consumer behavior, due to the high reliability and credibility transmitted by family and friends. In contrast, few studies have examined the interaction between perceived risk and eWOM source credibility (Hussain et al., 2017).

Notwithstanding the above, the review of the theoretical framework also revealed a gap in the literature on WOM credibility in situations involving multiple or many communicators and receivers and how this ultimately affects the end consumer. This would include, for instance, situations in which one person communicates a message to another, who acts as an intermediary, both receiving the original message and passing it along to a third party, i.e., the end consumer. In such cases, the original message can be altered or distorted, chipping away at the credibility of the WOM review as a source of information. This lends much more strength to written comments and reviews, such as eWOM, which can ultimately reduce risk and increase consumer confidence.

Another feature that distinguishes eWOM from traditional WOM is the speed with which it spreads and the ease of access to it. In this regard, when consumers need information about a product or service, they ultimately turn to online media (eWOM) for two reasons. First, they can get the information more quickly, as there is no need to wait for someone else – a friend or family member – to offer an opinion about what they wish to consume. Second, if they have already received WOM reviews, they can use eWOM to corroborate the information received. Therefore, credibility and speed are the two main features not only distinguishing the two media, but also influencing consumer behavior.

Finally, the analysis of the review showed that these two concepts – WOM and eWOM – while seemingly the same, are at the same time very different. The Internet has transformed traditional WOM into eWOM. The communication of opinions is no longer done interpersonally (i.e., person-to-person or face-to-face), but rather is mediated by ICT. However, the many studies conducted (e.g., Katz and Lazarsfeld, 1966; Brown et al., 2007; Daugherty and Hoffman, 2014; Yang, 2017) agree that they are the media most able to influence consumer behavior and the most often used to obtain information before, during, and after consuming a given product or service. For example, in the field of tourism, eWOM is considered the most influential pre-purchase source of travel information (Sotiriadis and Van Zyl, 2013).

## Author Contributions

This paper tries to offer a clearer understanding of the two concepts through a literature review and an exploration of how, as a result of advances in ICT, traditional WOM has given rise to eWOM. The author has made an important, direct, intellectual contribution to this paper and has approved it for publication.

## Conflict of Interest Statement

The author declares that the research was conducted in the absence of any commercial or financial relationships that could be construed as a potential conflict of interest.
